# Hair cortisol and changes in cortisol dynamics in chronic kidney disease

**DOI:** 10.3389/fendo.2024.1282564

**Published:** 2024-03-25

**Authors:** Laura Boswell, Arturo Vega-Beyhart, Miquel Blasco, Luis F. Quintana, Gabriela Rodríguez, Daniela Díaz-Catalán, Carme Vilardell, María Claro, Mireia Mora, Antonio J. Amor, Gregori Casals, Felicia A. Hanzu

**Affiliations:** ^1^ Endocrinology and Nutrition Department, Hospital Clínic de Barcelona, Barcelona, Spain; ^2^ Group of Endocrine Disorders, Institut d’Investigacions Biomèdiques August Pi Sunyer (IDIBAPS), Barcelona, Spain; ^3^ Endocrinology and Nutrition Department, Althaia University Health Network, Manresa, Spain; ^4^ Group of Nephrology and Transplantation, Institut d’Investigacions Biomèdiques August Pi Sunyer (IDIBAPS), Barcelona, Spain; ^5^ Nephrology Department, Hospital Clínic de Barcelona, Barcelona, Spain; ^6^ Department of Medicine, Faculty of Medicine and Health Sciences, University of Barcelona, Barcelona, Spain; ^7^ Biochemistry and Molecular Genetics Department, Hospital Clínic de Barcelona, Barcelona, Spain; ^8^ Centro de Investigación Biomédica en Red de Diabetes y Enfermedades Metabo´ licas Asociadas (CIBERDEM), Carlos III Health Institute, Madrid, Spain

**Keywords:** hair cortisol, salivary cortisol, dexamethasone suppression test, chronic kidney disease, visceral adipose tissue, cardiovascular risk

## Abstract

**Objective:**

We compared hair cortisol (HC) with classic tests of the hypothalamic–pituitary–adrenal (HPA) axis in chronic kidney disease (CKD) and assessed its association with kidney and cardiometabolic status.

**Design and methods:**

A cross-sectional study of 48 patients with CKD stages I–IV, matched by age, sex, and BMI with 24 healthy controls (CTR) was performed. Metabolic comorbidities, body composition, and HPA axis function were studied.

**Results:**

A total of 72 subjects (age 52.9 ± 12.2 years, 50% women, BMI 26.2 ± 4.1 kg/m^2^) were included. Metabolic syndrome features (hypertension, dyslipidaemia, glucose, HOMA-IR, triglycerides, waist circumference) and 24-h urinary proteins increased progressively with worsening kidney function (p < 0.05 for all). Reduced cortisol suppression after 1-mg dexamethasone suppression (DST) (p < 0.001), a higher noon (12:00 h pm) salivary cortisol (p = 0.042), and salivary cortisol AUC (p = 0.008) were seen in CKD. 24-h urinary-free cortisol (24-h UFC) decreased in CKD stages III–IV compared with I–II (p < 0.001); higher midnight salivary cortisol (p = 0.015) and lower suppressibility after 1-mg DST were observed with declining kidney function (p < 0.001). Cortisol-after-DST cortisol was >2 mcg/dL in 23% of CKD patients (12.5% in stage III and 56.3% in stage IV); 45% of them had cortisol >2 mcg/dL after low-dose 2-day DST, all in stage IV (p < 0.001 for all). Cortisol-after-DST was lineally inversely correlated with eGFR (p < 0.001). Cortisol-after-DST (OR 14.9, 95% CI 1.7–103, p = 0.015) and glucose (OR 1.3, 95% CI 1.1–1.5, p = 0.003) were independently associated with eGFR <30 mL/min/m^2^). HC was independently correlated with visceral adipose tissue (VAT) (p = 0.016). Cortisol-after-DST (p = 0.032) and VAT (p < 0.001) were independently correlated with BMI.

**Conclusion:**

Cortisol-after-DST and salivary cortisol rhythm present progressive alterations in CKD patients. Changes in cortisol excretion and HPA dynamics in CKD are not accompanied by significant changes in long-term exposure to cortisol evaluated by HC. The clinical significance and pathophysiological mechanisms explaining the associations between HPA parameters, body composition, and kidney damage warrant further study.

## 1 Introduction

Chronic kidney disease (CKD) is a condition affecting a substantial proportion of the general population; the overall prevalence of CKD stages III–V according to the Kidney Disease Improving Global Outcomes (KDIGO) guidelines ranges from 2% to 17%, with estimates of higher prevalence when including patients in earlier stages (abnormal urinary albumin excretion with preserved estimated glomerular filtration rate [eGFR]) (1). This condition is comorbid with diabetes, hypertension, and other metabolic complications and dramatically increases the risk of cardiovascular disease and premature mortality (2). Although a few studies have described changes in the hypothalamic–pituitary–adrenal (HPA) axis in the presence of CKD, evidence is scarce and previous studies have limitations: they are series with a low number of participants, two only include patients in haemodialysis, and all only performed a partial evaluation of the HPA axis (3–6).

Thus, the effect of CKD on the HPA axis and its mechanisms and possible consequences remain unknown. HPA axis evaluation is complex, and standard biochemical tests have limitations and methodologic difficulties (7). Also, they all have in common the measurement of cortisol in the acute (serum, saliva) or in a very short (24-h urine) timeframe, reflecting acute changes of the HPA axis but not mid- or long-term exposure (8, 9). Considering the changes described above and the unreliability of 24-h-urine cortisol when kidney function is impaired, HPA functional disturbances in CKD present a challenge for the differential diagnosis of pseudo-Cushing states and Cushing’s syndrome (CS) in this setting. Hence, new diagnostic strategies are required.

In recent years, the measurement of hair cortisol (HC) concentrations has emerged as a reliable strategy for quantifying long-term exposure to cortisol. As hair in the area of the posterior vertex grows at a relatively constant speed (1 cm/month), exposure to cortisol during the previous months can be estimated by measuring cortisol concentrations in this area (10, 11). Several studies have displayed evidence of its validity, test and retest reliability, and correlation with classical biochemical tests of the HPA axis (12, 13). It is a validated measure in the study of CS, especially in cyclic CS (14–16). There is also evidence that HC increases in acute and chronic stress situations (9), and it is widely used in psychopathology (17). HC is also predictive of metabolic syndrome (18), type 2 diabetes (T2D) and cardiovascular disease (19). Higher levels of HC have been described in some pseudo-Cushing states (alcoholism, obesity, depression). However, no studies have evaluated HC in CKD.

Against this background, we aimed to study HC in the setting of CKD, compare it with the classical biochemical tests of the HPA axis, and investigate its association with kidney function outcomes and cardiometabolic status.

## 2 Methods

### 2.1 Study design and participants

A cross-sectional, controlled, single-centre study was performed. Patients had to fulfil criteria for chronic kidney disease (CKD): decreased eGFR through the Chronic Kidney Disease Epidemiology Collaboration [CKD-EPI] equation and/or kidney damage as defined by structural or functional abnormalities other than decreased eGFR (p. ex. albuminuria ≥30 mg/g, structural abnormalities detected by imaging) for >3 months according to the KDIGO guidelines (20). Patients with CKD stages I–II (eGFR >60 mL/min/1.73 m^2^), stage III (eGFR 30–60 mL/min/1.73 m^2^), and stage IV (eGFR 15–30 mL/min/1.73 m^2^) CKD with preserved diuresis were prospectively included and matched with healthy controls by age, sex, and body mass index (BMI). Controls had to fulfil both criteria of normal kidney function: eGFR >60 mL/min/1.73 m^2^) and absence of kidney damage markers. The age ranges for CKD patients and healthy controls were similar: 54 (45–61.3) years (median (interquartile range [IQR]) and 23.4–77.4 years (minimum and maximum) for CKD patients and 54.6 (47.9–63.8) years (median [IQR]) and 27.2–69.2 years (minimum and maximum) for control subjects.

CKD subjects were divided according to stages (mild: stages I–II, moderate: III and severe: IV) to be able to describe the differences in HPA axis physiology according to CKD severity. A control group with preserved kidney function and no structural kidney damage was included for a normality reference.

All subjects had preserved diuresis (defined as absence of oliguria or anuria). Subjects with CKD all had a urinary output >1,000 mL/24 h and controls >800 mL/24 h.

Patients were prospectively included from the cohort of CKD patients who visited the Nephrology Department of the Hospital Clínic de Barcelona between December 2019 and June 2022. Controls were selected from among hospital workers or their acquaintances, CKD patients’ spouses, and volunteers. Controls were initially selected based on medical records and underwent the same screening and inclusion/exclusion criteria as CKD subjects to ensure they had no kidney damage whatsoever (eGFR >60 mL/min/1.73 m^2^) and an absence of kidney damage markers).

The exclusion criteria were active glucocorticoid, immunosuppressive or anti-inflammatory treatment, diabetes mellitus, morbid obesity (BMI ≥40 kg/m^2^), drugs interfering with the HPA axis, active malignancy, active Cushing’s syndrome, or pseudo-Cushing states such as depression or high-risk alcohol consumption. The rationale behind these exclusion criteria was to rule out any drug interference or physiological or pathophysiological alteration of the HPA axis unrelated to CKD.

The study protocol was conducted according to the principles of the Declaration of Helsinki and approved by the Institution’s Research Ethics Committee (reference number: HCB/2019/0845). All participants provided written informed consent.

### 2.2 Study outline

**Figure 1:**
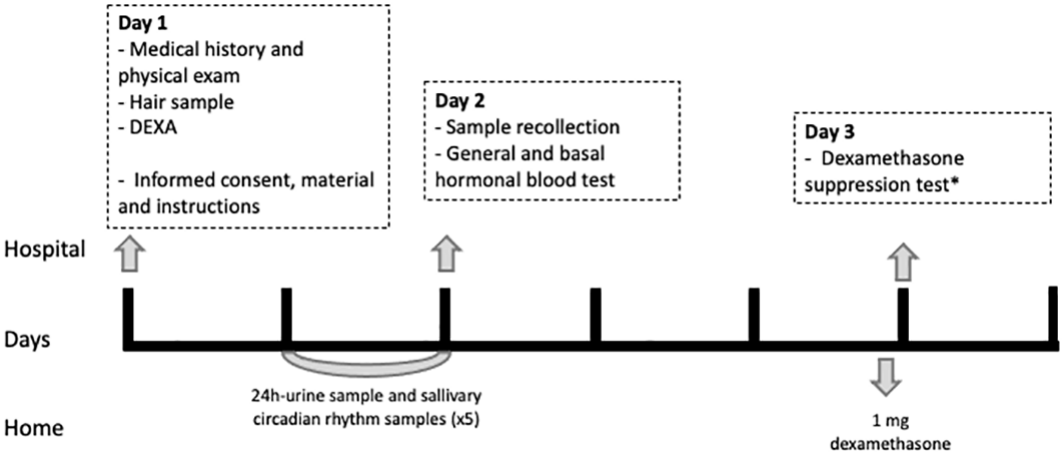
Study outline. DEXA, dual-energy X-ray absorptiometry (DEXA). *Those participants with a cortisol after 1-mg DST >2 mcg/dL underwent a low-dose 2-day DST (2 mg/6h).

The subjects underwent clinical and analytical assessment of metabolic comorbidities, body composition analysis using dual-energy X-ray absorptiometry (DEXA), and a thorough evaluation of the HPA axis. On the first appointment, all subjects (both CKD patients and controls) were screened for inclusion and exclusion criteria and signed the informed consent. Then, a thorough clinical and body composition evaluation was performed and the hair sample was collected. Finally, the instructions and material to collect the saliva and 24-h urine samples were handed over. On the second appointment, the subjects had an early-morning blood test and handed over the samples. On the last appointment, the dexamethasone suppression test was performed. The time interval between the first and second appointments was 4 (2–14) days, and that between the second and last appointments was 10 (4–21) days. The study outline is shown in **Figure 1**.

#### 2.2.1 Clinical and body composition evaluation

At inclusion, all subjects underwent a complete clinical evaluation. Age, sex, smoking status, metabolic comorbidities, and current medical treatment were recorded for all participants. CKD stage, aetiology, duration, and previous treatments were recorded for CKD patients. Both the patients and controls were screened for pseudo-Cushing states. Data regarding hair colour, treatment, and frequency of washing were also recorded. Metabolic comorbidities were defined as follows: hypertension was defined as repeated clinical systolic blood pressure (BP) ≥140 mmHg and/or diastolic BP ≥90 mmHg or active treatment with antihypertensive drugs; dyslipidaemia was defined as LDL-cholesterol (LDL-c) >160 mg/dL, low high-density lipoprotein cholesterol (HDL-c) (<40 mg/dL in men and <45 mg/dL in women), triglycerides ≥150 mg/dL, or active treatment with lipid-lowering drugs (statins, ezetimibe, or fibrates); and prediabetes was defined as fasting plasma glucose 100 mg/dL to 126 mg/dL or HbA_1c_ 5.7% to 6.4% and obesity as a BMI ≥30 kg/m^2^). Anthropometric parameters (weight, height, BMI, blood pressure, and waist and hip circumferences) were obtained by physical examination as previously described (21–23).

Body composition (quantification and distribution of fat mass) was studied using DEXA, an indirect and non-invasive technique (*iDEXA, General Electrics*). DEXA was used to measure total fat mass, fat distribution (proportion of android and gynoid fat), subcutaneous adipose tissue, and visceral adipose tissue (VAT).

#### 2.2.2 HPA axis evaluation

##### 2.2.2.1 Chronic exposition to cortisol: hair cortisol concentration

HC was chosen because it is the only available test able to quantify cortisol levels in the mid-long term. Hair samples were processed and analysed for cortisol following the method previously described by Sauvé et al. (24) In the first visit, a hair sample of >1-cm length from the posterior vertex was obtained. As hair in this area grows at a relatively constant speed (1 cm/month), cortisol exposure during previous months can be estimated (10, 11). Once the sample had been cut with metal scissors, as close as possible to the scalp, a thread was tied to the end to signal the external tip. The samples were kept in an envelope, protected from light and at room temperature until the analysis. At the moment of evaluation, they were placed in a container and washed twice with 10 mL isopropanol to eliminate possible contaminants. The proximal 1 cm was cut and analysed as representative of the mean cortisol value in the last month. Prior to the cortisol extraction, the hair was cut into small fragments using surgical scissors and weighed to obtain the hair mass (minimum 25 mg). Cortisol extraction was performed by adding 1 mL of methanol to the glass vial where the hair was weighed and incubated overnight with slow shaking. Then, the supernatant was transferred to an assay tube and evaporated with nitrogen. Finally, the remnant was resuspended with the diluent of the cortisol assay and agitated until complete dissolution. Cortisol was measured by ELISA (Salimetrics LLC, State College, PA, USA). The weighing, extraction, and analysis of the same hair sample collection were performed in duplicate when a sufficient sample was available and the mean coefficient of variation of duplicates was 8%. Interassay coefficient of variation was 7%.

##### 2.2.2.2 Cortisol circadian rhythm and overall cortisol secretion analysis/study

Saliva samples were collected at regular intervals during the day to assess cortisol circadian rhythm and to indirectly estimate cumulative cortisol exposure during the day. Furthermore, evening and late-night cortisol measurements are more appropriate to detect high cortisol exposure when a mild corticoid excess is suspected. Moreover, salivary cortisol measures free biologically active cortisol, which is less altered by binding protein levels than serum cortisol. 24-h UFC was measured to estimate the cortisol excreted in a 24-h period. Basal cortisol and ACTH were measured to establish the cortisol level at its diurnal peak and the central activity of the HPA axis, respectively (25). Cortisol-binding globulin (CBG) and albumin (see section 2.2.3) were measured to assess levels of circulating cortisol-binding proteins.

The subjects collected a 24-h urine sample and five saliva samples (8:00 h am, 12:00 h pm, 4:00 h pm, 8:00 h pm, 12:00 am) from the day before. During the first visit, they systematically received instructions for adequate sample collections to minimise variability in sample collection. If instructions had not been carefully followed, subjects were reinstructed and sample collection was performed once again. Furthermore, adequate 24-h urine recollection was verified through 24-h urinary creatinine levels.

Serum and EDTA plasma samples were obtained in the early morning (8:00 h am) after a 12-h fast to measure basal cortisol and ACTH, respectively, as well as metabolic parameters (see below).

The samples were kept at −80°C until analysis. Hormonal analyses were performed in the Hormonal Laboratory, applying the standard procedures, with the following methods: 24-h UFC by chemiluminescence immunoassay (Liaison; DiaSorin, Saluggia, Italy) after extraction with dichloromethane; serum cortisol was measured by chemiluminescence immunoassay (Atellica IM 1600, Siemens Healthineers, Tarrytown, NY, USA); ACTH was measured using chemiluminescence immunoassay (IMMULITE 2000; Siemens Healthineers, Tarrytown, NY, USA); CBG was measured by radioimmunoassay (DiaSource, Louvain-la-Neuve, Belgium); and salivary cortisol was measured with a specifically validated ELISA (Salimetrics LLC, State College, PA, USA).

##### 2.2.2.3 Dynamic study of the HPA axis: dexamethasone suppression test

The DST was performed to study the HPA axis negative feedback mechanism. We expected a certain degree of resistance after the DST in advancing CKD and an adequate suppression after a low-dose 2-day-DST in all subjects (5). Dexamethasone at the doses given to perform the functional tests has rarely any side effects. Nevertheless, all subjects were questioned for allergy to the active principle in the first evaluation.

The cortisol from the first blood test was used as the basal cortisol. On the first visit, subjects were given a 1-mg dexamethasone pill and instructions on when to take it. The subjects took the dexamethasone pill at 11:00 h pm the night before the second blood test and were instructed to follow a 12-h fast. The following morning (8:00–9:00 h am), a serum sample to measure (post-DST) cortisol was obtained.

Those subjects with cortisol >2 mcg/dL after the DST went through a low-dose 2-day-DST: a blood test for basal (8:00–9:00 h am) cortisol was extracted on the first day, the subject took 0.5 mg every 6 h for 48 h, and on the third day, a blood test for post 2-day-DST cortisol was performed (8:00–9:00 h am).

#### 2.2.3 Metabolic and systemic inflammation evaluation

The basal blood and 24-h urine samples were analysed in the local laboratory using standardised assays. Specifically, glucose, creatinine, sodium, potassium, calcium, magnesium, total cholesterol, HDL-c, triglycerides, and high-sensitive C-reactive protein (hs-CRP) were measured in serum on an Atellica CH930 chemistry analyser (Siemens Healthcare Diagnostics). LDL-c was calculated using the Friedewald formula. Serum and 24-h urinary creatinine, proteins, and albumin were measured on an Atellica CH930 chemistry analyser. Insulin was measured by chemiluminescence immunoassay on an Atellica IM1 600 analyser, and homeostatic model assessment-insulin resistance (HOMA-IR) was calculated using the following formula: glucose (mmol L^−1^) × insulin (mIU L^−1^)/22.5. HbA_1C_ was measured on a Tosoh G8 HPLC Analyzer. Leukocyte formula and blood count were measured on an ADVIA 120 haematology system (Siemens Healthcare Diagnostics).

### 2.3 Statistical analyses

Data are presented as median and 25th and 75th percentiles (IQR), mean ± SD or number (percentage). Normal distribution of continuous variables was evaluated with the Kolmogorov–Smirnov test. Between-group differences in clinical, anthropometric, and laboratory variables were assessed using unpaired Student’s t-test, Mann–Whitney test, and chi-square tests as appropriate, for parametric, non-parametric, and categorical values, respectively. Multiple-group analyses were performed using ANOVA or Kruskal–Wallis as appropriate. Bonferroni and Jonckheere–Terpstra were performed to test for multiple comparisons after one-way ANOVA and Kruskal–Wallis, respectively. To examine linear trends, the Mantel–Haenszel test for categorical variables and the linear contrast analysis for continuous variables were used.

Univariate linear regression analyses were performed to evaluate the association of hair cortisol and cortisol after DST with kidney function and cardiometabolic variables (age, waist-to-hip ratio (WHR), eGFR, glucose, and VAT volume). Variables with a significant association were then included in stepwise multiple linear regression models to identify independent parameters related to hair cortisol and cortisol after DST. Multivariate linear regression models were adjusted for age, sex, BMI, and clinical data or conventional lab parameters that resulted in statistical significance when performing the univariate correlation tests: Particularly, the first model included eGFR, cortisol after DST, age, BMI, and smoking; the second model included cortisol after DST, glucose, eGFR, age, BMI, hypertension, CKD duration, LDL-c, and hs-PCR, and the third model included HC, VAT volume, age, BMI, smoking, sex, and eGFR.

Significance level was defined as a p-value <0.05. IBM SPSS Statistics 23.0 (SPSS, Inc; Chicago, Illinois) was used to perform the statistical analysis.

## 3 Results

### 3.1 Study population characteristics and cardiometabolic status according to CKD stage

A total of 72 subjects were included: 48 patients with CKD (16 in stages I–II, 16 in stage III, and 16 in stage IV) and 24 paired controls; age 52.9 ± 12.2 years (minimum: 23.4 years, maximum: 77.4 years), 50% women, and BMI 26.2 ± 4.1 kg/m^2^ (minimum: 19.3 kg/m^2^, maximum: 37.4 kg/m^2^).

The aetiology of CKD was the following: 27 (56.3%) polycystic kidney disease, 7 (14.6%) IgA nephropathy, 4 (8.3%) hypertensive nephroangiosclerosis, 4 (8.3%) focal segmental glomerulosclerosis, and 6 (12.5%) other causes. There were no subjects with diabetic CKD or glomerulopathies requiring corticoid treatment (such as lupic nephropathy) in this study, as diabetes and glucocorticoid treatment were both exclusion criteria. Hence, only CKD aetiologies which were compatible (either because of their physiopathology or their treatment) with the study aims and which would not hamper the HPA axis measurements were included. In the general population, diabetic CKD is the main cause of CKD (approximately 30%–50%) and glomerulopathies are infrequent (26).

In CKD patients, a stepped increase in hypertension and dyslipidaemia prevalence, lipid-lowering therapy, and increasing levels of glucose, insulin, HOMA-IR, triglycerides, and 24-h urinary protein excretion were observed with worsening kidney function (p < 0.05 for all) whereas no changes were seen in other lipid parameters. Lipid profiles excluding triglycerides were similar despite an increasing prevalence of dyslipidaemia. CKD subjects were much more frequently on lipid-lowering drugs (75% of CKD subjects and 33.3% controls, p = 0.027). Moreover, not only were there more subjects receiving these drugs in more advanced kidney stages (p for trend <0.001) but also the proportion of subjects with dyslipidaemia receiving therapy was higher (p for trend = 0.003). No differences were observed regarding hs-CRP, all subjects had low levels.

Moreover, systolic and diastolic BP increased with advancing CKD stage (p < 0.05). BP was clinically significantly higher in the stage III and IV groups, which is consistent with a prevalence of hypertension approximately 90% in these subjects.

Visceral fat was assessed through clinical surrogates (such as waist circumference [WC] and WHR) and measured with DEXA. An increased WC was observed in stage III CKD subjects (p = 0.007) compared with controls and those in stages I–II; a non-significant trend towards increasing WC with advancing CKD stage was also seen (p = 0.059). WHR was high in all groups but significantly higher in stage III (p = 0.030). A trend to an increased VAT volume and mass measured with DEXA (p = 0.073) was observed with advancing CKD stage.

A significative linear trend for cardiovascular disease (CVD) (p = 0.042) was also observed: Three subjects had CVD, all in CKD stages III–IV. Cardiometabolic factors according to CKD stages and controls are presented in **Table 1**.

**Table 1:** Study population characteristics and metabolic changes by CKD stage.

	Controls (n = 24)	Stages I–II (n = 16)	Stage III (n = 16)	Stage IV (n = 16)	p	p for trend
**Age (years)**	52.7 ± 12.9	47.5 ± 13	53.6 ± 8.8	57.8 ± 11.8	0.121	0.145
**Sex (women)**	12 (50)	9 (56.3)	8 (50)	7 (43.8)	0.919	0.685
**BMI (kg/m^2^)**	25.6 ± 4.6	24.9 ± 3.2	28.3 ± 4.1	26.2 ± 11.8	0.101	0.213
Summary of key findings						
**Hypertension**	8 (33.3)	8 (50)	14 (87.5)	15 (93.8)	<0.001	<0.001
**Dyslipidaemia**	9 (37.5)	3 (18.8)	8 (50)	12 (75)	0.012	0.004
Lipid-lowering therapy* **Proportion of the total** **Proportion of subjects with dyslipidaemia**	3 (12.5) 3 (33)	1 (6.3) 1 (33.3)	6 (37.5) 6 (75)	11 (68.8) 11 (91.7)	<0.001 0.024	<0.001 0.003
**Glucose (mg/dL)**	85 (77-92)	82 (77-87)	82 (78-86)	92 (84-99)	0.027	0.108
**Insulin (mU/L)**	7.3 (4.9-8.5)	7.3 (4.8-8.6)	10.5 (6.6-18.9)	11.4 (7.7-13.5)	0.004	0.002
**HOMA-IR**	1.45 (0.92-2.11)	1.48 (0.95-1.85)	2.12 (1.31-4.31)	2.38 (1.6-3.34)	0.007	0.003
**Triglycerides (mg/dL)**	86 (72-110)	89 (73-109)	111 (88-164)	135 (102-210)	0.005	0.001
**Albuminuria (mg/24 h)**	0 (0-4)	20 (8-43)	64 (16-216)	520 (38-1066)	<0.001	<0.001
Kidney function						
**eGFR (mL/min/m^2^)**	90 (85-90)	90 (77-90)	37 (34-51)	24 (20-27)	<0.001	<0.001
**Diuresis volume (mL/24 h)**	1750 (1200-2100)	1900 (1500-2475)	2000 (1725-2275)	2363 (1919-3225)	0.029	<0.001
**Urinary creatinine (mg/24 h)**	1187 ± 301	1223 ± 461	1163 ± 236	1197 ± 329	0.993	0.001
Metabolic assessment						
**Clinical comorbidities**						
**Active smokers**	0 (0)	4 (25)	3 (18.8)	1 (6.3)	0.059	0.452
**Number of antihypertensive drugs**	1 (1-1.8)	1.5 (1-2)	2 (1-2.3)	1 (1-2)	0.480	0.345
**Family history of premature CVD**	4 (16.7)	1 (6.3)	2 (12.5)	3 (18.8)	0.735	0.845
**CVD**	0 (0)	0 (0)	1 (6.3)	2 (12.5)	0.195	0.042
**SBP (mmHg)**	123 ± 18	127 ± 17	131 ± 11	140 ± 16	0.016	0.002
**DBP (mmHg)**	79 ± 10	83 ± 6	84 ± 10	85 ± 12	0.194	0.039
**Waist circumference (cm)**	95 ± 15	94 ± 5	106.5 ± 9	98 ± 9	0.007	0.059
**Waist-to-hip ratio**	0.97 (0.89-0.99)	0.94 (0.90-0.97)	0.99 (0.95-1.04)	0.97 (0.93-1-.02)	0.030	0.146
Laboratory measures						
**HbA1c (%)**	5.5 ± 0.4	5.4 ± 0.35	5.4 ± 0.37	5.7 ± 0.46	0.088	0.149
**Total cholesterol (mg/dL)**	199 ± 36	189 ± 35	198 ± 42	199 ± 41	0.848	0.897
**LDL-c (mg/dL)**	128 ± 28	116 ± 29	116 ± 31	121 ± 34	0.531	0.387
**HDL-c (mg/dL)**	53 ± 13	56 ± 11	56 ± 18	49 ± 13	0.472	0.487
**hs-CRP (mg/dL)**	0.12 (0.04-0.28)	0.11 (0.04-0.30)	0.27 (0.09-0.64)	0.14 (0.07-0.39)	0.285	0.217
**Albumin (g/L)**	45 (44-46)	44 (43-45)	45 (44-48)	44.5 (42-47)	0.225	0.862
Body composition**						
**Total fat mass (%)**	32.7 ± 6.5	32.1 ± 10.3	36.5 ± 5.8	33.3 ± 6.1	0.306	0.406
**VAT (cm** ^3^ **)**	979.5 (132.5-2222)	663.5 (203-841)	978 (648-1648)	1322 (722-1935)	0.073	0.154
**VAT (g)**	924 (124.8-2095.8)	626.5 (191.3-794.3)	923 (612-1554)	1248 (681-1827)	0.074	0.157

Values expressed as mean ± SD, median (interquartile range) or number (percentage). p-value according to ANOVA or Kruskal–Wallis are represented.

*Active treatment with statins or ezetimibe.

**Measured with dual-energy X-ray absorptiometry (DEXA).

BMI, body mass index; CVD, cardiovascular disease; DBP, diastolic blood pressure; eGFR, estimated glomerular filtration rate; HDL-c, high-density lipoprotein cholesterol; HOMA-IR, homeostatic model assessment-insulin resistance; LDL-c, low-density lipoprotein cholesterol; SBP, systolic blood pressure; TC, total cholesterol; hs-PCR, high-sensitivity C-reactive protein; VAT, visceral adipose tissue.

### 3.2 Hair cortisol determinants

Hair cortisol was positively correlated with age (β 0.835, standardised-β 0.279, p = 0.018), but this association was lost when adjusting for confounders. No differences in hair cortisol were seen according to sex (p = 0.287), smoker status (p = 0.979), or smoking index (p = 0.420). Hair cortisol was correlated with alcohol consumption (measured as standard drinks, 10 g of alcohol) (β 1.812, standardised-β 0.248, p = 0.036). Nevertheless, this finding was not clinically relevant as alcohol consumption was low in all subjects. None had a high-risk alcohol consumption as this was an exclusion criterion of the study. Hair cortisol was unrelated to the frequency of hair washing (p = 0.579), hair colour (p = 0.909), or hair treatment (p = 0.346).

### 3.3 Hypothalamic–pituitary–adrenal axis study

First, changes in the HPA axis of the whole CKD population compared with controls were analysed. A reduced cortisol suppression after DST (1.3 vs. 0.9 µg/dL, p < 0.001), a higher noon (12:00 h pm) salivary cortisol (p = 0.042), and a higher salivary cortisol area under the curve (AUC) (p = 0.008) were seen in patients with CKD compared with controls.

A trend to a lower 24 h UFC was also observed. Hair cortisol, ACTH, cortisol, CBG, and the remaining salivary cortisol rhythm points remained unchanged.

Then, the HPA axis was studied according to CKD severity: 24-h UFC was decreased in stages III–IV compared with CKD stages I–II and controls (p < 0.001), despite an increased 24-h urinary volume.

**Figure 2:**
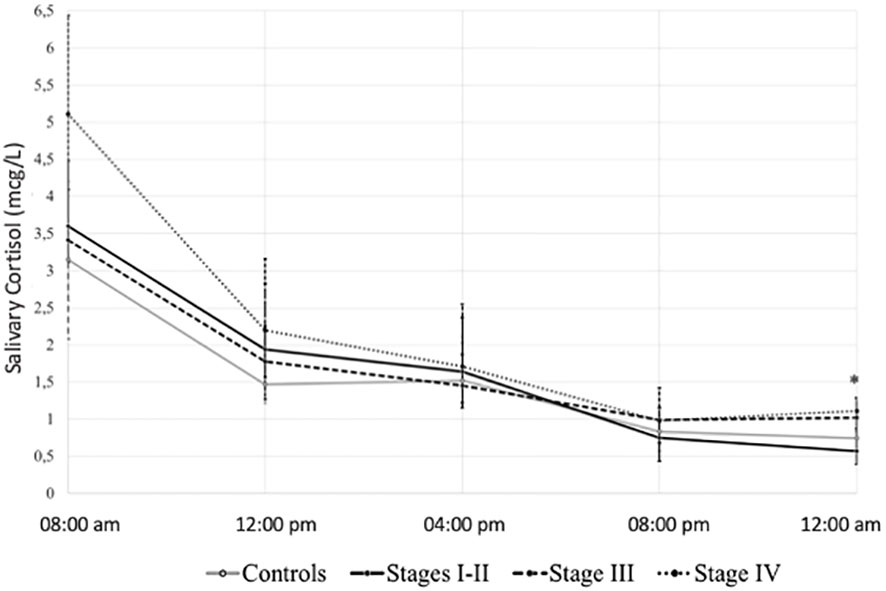
Salivary cortisol 24-h rhythm according to study group. *p value <0.05. Salivary cortisol (mcg/L) in median (interquartile range).

Higher late-night (12:00 h am) salivary cortisol levels (p = 0.015) and a significative linear trend for salivary cortisol AUC (p = 0.006) were observed with declining kidney function (**Figure 2**).

**Figure 3:**
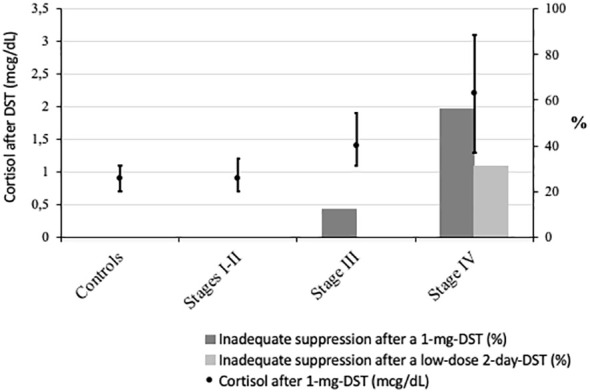
Cortisol after dexamethasone suppression test (DST) across study groups. points: cortisol after 1-mg DST (mcg/dL) in median (interquartile range); dark bars: % of subjects with cortisol after 1-mg DST >2 µg/dL; light bars: % of subjects with cortisol after a low-dose 2-day DST (2 mg/6h) >1.8 µg/dL. p value for all <0.001.

A lower suppressibility after a 1-mg DST was observed with declining kidney function (p < 0.001). Among all, 11 (23%) patients with CKD had post-DST cortisol of >2 mcg/dL (none in controls or stages I–II, 2 [12.5%] in CKD stage III, and 9 [56.3%] in the stage IV group, p < 0.001). To further assess resistance to dexamethasone suppression, these subjects underwent a low-dose 2-day DST (2 mg/6 h): 5 (45.5%) still had an unsuppressed cortisol (>1.8 mcg/dL) after the test, all in stage IV. Namely, the two subjects in stage III with an increased cortisol after DST suppressed correctly after a 2-day DST whereas only 4/9 (44.4%) did so in stage IV (p < 0.001 for all) (**Figure 3**). Thus, negative feedback regulation is progressively impaired with declining kidney function.

Post-DST cortisol was correlated with 08:00 pm salivary cortisol in controls (r = 0.533, p = 0.016) and in patients with CKD stages III (r = 0.469, p = 0.046) and IV (r = 0.811, p < 0.001). However, no correlation was found with late-night (12:00 h am) salivary cortisol in any group. No differences were observed in HC, ACTH, basal serum cortisol, CBG, or salivary cortisol in the remaining time frames (**Table 2**).

**Table 2:** HPA axis changes according to CKD stage.

	Controls (n = 24)	Stages I–II (n = 16)	Stage III (n = 16)	Stage IV (n = 16)	p	p for trend
Chronic exposure to cortisol						
**Hair cortisol (pg/mg)**	3.95 (2.40-5.08)	2.90 (2.13-3.83)	4 (2.88-5.38)	3.55 (2.78-4.75)	0.263	0.463
Cortisol circadian rhythm						
**ACTH (pg/mL)**	20 (12.3-28)	21 (18.3-25.6)	23.5 (17.5-29.3)	25 (15-28)	0.525	0.188
**Cortisol (mcg/dL)**	16 ± 4	18.4 ± 3.5	16.2 ± 4.7	17.3 ± 5.2	0.342	0.350
**CBG (mcg/mL)**	52.3 ± 9.8	49.3 ± 7	53.3 ± 11.1	52.5 ± 6.9	0.601	0.144
**24 h UFC (mcg/24 h)**	54.4 (34.8-74.4)	54 (42.3-83.4)	31.8 (24.2-43.3)	30.8 (21-46)	<0.001	<0.001
**Diuresis volume (mL/24 h)**	1750 (1200-2100)	1900 (1500-2475)	2000 (1725-2275)	2363 (1919-3225)	0.029	<0.001
**8:00 am-salivary cortisol (mcg/L)**	3.15 (2.59-4.20)	3.60 (3.17-4.48)	3.41 (2.08-4.09)	5.11 (3.11-6.44)	0.130	0.092
**12:00 pm-salivary cortisol (mcg/L)**	1.47 (1.21-1.96)	1.94 (1.57-2.25)	1.78 (1.47-2.83)	2.20 (1.27-3.16)	0.235	0.070
**4:00 pm-salivary cortisol (mcg/L)**	1.52 (1.22-2.03)	1.64 (1.15-2.37)	1.45 (1.22-1.87)	1.71 (1.22-2.55)	0.848	0.515
**8:00 pm-salivary cortisol (mcg/L)**	0.83 (0.57-0.99)	0.75 (0.43-0.99)	0.99 (0.73-1.15)	0.98 (0.68-1.42)	0.131	0.073
**12:00 am-salivary cortisol (mcg/L)**	0.74 (0.58-0.83)	0.57 (0.39-0.87)	1.02 (0.74-1.29)	1.11 (0.74-1.29)	0.015	0.014
Salivary cortisol AUC (mcg*24h/L)	37.98 ± 8.16	42.19 ± 14.73	44.54 ± 18.55	50.88 ± 15.38	0.138	0.006
Dynamic study of the HPA axis						
**Cortisol after DST (µg/dL)**	0.9 (0.7-1.1)	0.9 (0.7-1.2)	1.4 (1.1-1.9)	2.2 (1.3-3.1)	<0.001	<0.001
**Cortisol after DST >1.8 µg/dL**	0 (0)	0 (0)	4 (25)	9 (56.3)	<0.001	<0.001
**Cortisol after DST >2 µg/dL**	0 (0)	0 (0)	2 (12.5)*	9 (56.3)**	<0.001	<0.001

Values expressed as mean ± SD, median (interquartile range) or number (percentage). p-value according to ANOVA or Kruskal–Wallis are represented.

*100% had a normal response (cortisol ≤1.8 µg/dL) after a low-dose 2-day DST (2 mg/6 h).

**45% had a normal response to a low-dose 2-day DST (2 mg/6 h).

ACTH, adrenocorticotropic hormone; AUC, area under curve; CKD, chronic kidney disease; CBG, cortisol-binding-globulin; DST, 1-mg dexamethasone suppression test; HPA, hypothalamus–pituitary–adrenal; UFC, urinary-free cortisol.

In the whole cohort, eGFR held an independent inverse association with cortisol after DST adjusted for confounders (age, BMI, smoking), which explained 48% of the variance in the serum levels in patients (**Table 3**). Correlation factors of the remaining clinical and biochemical variables and cortisol after DST can be found on **Supplementary Table 2**.

**Table 3:** Independent parameters associated with serum cortisol levels after DST.

Parameter	Pearson correlation	Degree of association with cortisol after DST of effect				
		R^2%^	β	95% CI		p value
				LL	UL	
eGFR (CKD-EPI)	-0.661*	48.1	-0.210	-0.310	-0.011	<0.001
Smoking*	0.566*	14.0	0.020	-0.007	-0.033	0.004
Sex	-0.210	–	-0.466	-1.157	0.226	0.179
BMI	0.021	–	-0.014	-0.041	0.013	0.297
Age	-0.202		-0.147	-0.498	0.009	0.093

*p value <0.05 in univariate testing. R^2^% indicates the percent variation in the concentration of cortisol after DST explained by the indicated factor. The β coefficient results from a standardised stepwise model. 95% CI: confidence intervals for coefficient β. p value: after Bonferroni correction to adjust for multiple testing.

BMI, body mass index; DST, 1-mg dexamethasone-suppression test; eGFR, estimated glomerular filtration rate; LL, lower limit; UL, upper limit.

Cortisol after DST (OR 14.9, 95% CI 1.7–130, p = 0.015) and glucose (OR 1.3, 95% CI 1.1–1.5, p = 0.003) were also independently associated with an eGFR <30 mL/min/m^2^, adjusted for confounders (age, BMI, hypertension, CKD duration, LDL-c, and hs-PCR) (**Supplementary Figure 1**).

### 3.4 Cardiometabolic and body composition status according to HPA changes

In unadjusted regression analyses, HC was linearly correlated with VAT volume (β 78.2, standardised-β 0.316, p = 0.007), WHR (β 13.8, standardised-β 0.258, p = 0.029), and age (see 3.3). Cortisol after DST was correlated with age (β 4.831, standardised-β 0.351, p = 0.004), WHR (β 3.6, standardised-β 0.298, p = 0.014), and glucose (β 3.034, standardised-β 0.266, p = 0.030) but not with VAT volume. Neither HC nor cortisol after DST correlated with BMI.

In multivariate regression analyses, HC (β 54.488, standardised-β 0.22, p = 0.015) was independently correlated with VAT volume, adjusted for age, BMI, smoking, sex, and eGFR (**Supplementary Table 1**).

Finally, in unadjusted regression analyses, a positive correlation between inflammation markers (neutrophil count and hs-PCR) and ACTH as well as between neutrophils and noon-salivary-cortisol was found, but the relation was lost when adjusted for confounders (data not shown).

## 4 Discussion

This is the first study to perform a thorough evaluation of the HPA axis including HC concentrations in subjects with CKD stages I to IV. We describe for the first time that chronic exposure to cortisol (through HC concentrations) remains unchanged in CKD, in contrast with changes in the cortisol circadian rhythm and dynamic study of the HPA axis. We describe a resistance to dexamethasone suppression and higher noon-salivary cortisol in CKD subjects compared with healthy controls, as well as increasing midnight-salivary cortisol and cortisol after DST and decreasing 24-h UFC as the kidney function worsens. Additionally, this is the first study to describe an independent association of HC with VAT volume. In the light of our findings, more data are needed to support the role of HC in the challenging differential diagnosis when hypercortisolism is suspected in the presence of CKD.

The HPA axis in CKD has seldom been studied. In the 1980s, a small study in end-stage CKD (haemodialysis) described increased free and total plasma cortisol, both at 8:00 h am and after a 1-mg DST as well as increased midnight mean plasma cortisol (3). Three decades later, another study in haemodialysis patients showed a preserved circadian rhythm, albeit with higher midnight nadir (plasmatic and salivary) cortisol and plasmatic ACTH (4). A few studies have studied the HPA axis in earlier stages. One study in CKD stages I–IV described an inverse correlation between midnight-salivary cortisol and eGFR. In this study, 10% had a false positive result after a DST (inadequate suppression), all with eGFR <90 mL/min/1.73 m^2^ and a normal response to a 2-day 2-mg DST. There were no changes in dexamethasone absorption or in transport proteins (5). Another study reported an increased concentration of both early morning cortisol and cortisol after DST, with similar ACTH, in patients with adrenal incidentalomas and moderately impaired renal function (eGFR <60 mL/min/1.73 m^2^) (6). In addition, a study including CKD stages I–IV described similar basal cortisol and greater ACTH compared with controls. Also, progressive lower morning UFC with decreasing eGFR (especially when eGFR was <29 mL/min) and a normal response to DST (27).

In line with these previous studies (28), in our cohort, a proportional resistance to DST was found with worsening kidney function (increasing cortisol after DST as well as the proportion of patients with inadequate suppression after DST—**Figure 3**). A previous pharmacokinetic study performed in advanced CKD in haemodialysis subjects revealed that 75% did not suppress after a 1-mg DST and 2-day DST but suppressed adequately after higher doses of oral or iv dexamethasone, suggesting a prolonged cortisol half-life (28).

In our study 45% of subjects with inadequate suppression after a 1-mg DST failed to normally suppress after a 2-day DST, all in stage IV CKD, revealing an even greater resistance to dexamethasone in this group. This contrasts with the study by Cardoso et al. (5) Although the average eGFR across groups was similar in both studies, our cohort included a higher proportion of patients with lower eGFR in the stage IV group. Other unmeasured factors of chronic stress could also explain this difference. Considering these results, the cutoff value for the DST should be revised to avoid false-positive results when eGFR is <60 mL/min/1.73 m^2^. Recently, a clinical trial has suggested that 3.2 µg/dL has the best diagnostic accuracy for patients with CKD and eGFR <30 mL/min/1.73 m^2^ (https://clinicaltrials.gov/ct2/show/NCT05568602). In our cohort, 100% of subjects with CKD stages I–III and 81.3% in stage IV had cortisol after DST below this cutoff.

Midnight salivary cortisol increased with worsening kidney function suggesting a progressive impairment in the circadian rhythm. This reproduces the findings by Cardoso et al. (5) We observed a tendency toward higher midnight salivary cortisol, a significant increase in noon-salivary cortisol, and an increasing salivary cortisol AUC with advancing CKD, suggesting a global, albeit discrete, increased exposure to cortisol during the 24 h. Previous studies in haemodialysis also described a higher salivary cortisol levels between noon and midnight (4), and a higher mean 24-h plasma cortisol compared with controls (3).

Low eGFR is a well-known condition for a false negative 24-h UFC (8, 27). In our cohort, only patients with preserved diuresis were included. We observed decreasing 24-h UFC with lowering eGFR (especially <60 mL/min/m^2^) despite a progressive increase in urinary output. This highlights the unreliability of 24-h UFC even when urinary output is within the normal/high range. Specifically, 24-h UFC is a source of false negative results when evaluating suspected hypercortisolism in CKD patients.

Early-morning cortisol and ACTH remained unchanged in CKD compared with controls. Existing literature is contradictory: In haemodialysis patients, an increase in basal cortisol and ACTH (3) was described in one article and no changes in basal but higher nadir cortisol and ACTH in the other (4). In earlier stages, a higher basal cortisol level was described (6) in one study. Regarding ACTH, one study described greater levels in CKD (27) whereas two studies did not report changes in basal ACTH (5, 6). A hypothesis is that the dynamics of the HPA axis would be progressively impaired with decreasing kidney function: first, night (nadir) levels increase, progressively earlier hours of the day are impaired, whereas basal levels remain the last hurdle. Blunted diurnal decline (higher evening salivary cortisol) seems to appear parallel to an impaired negative cortisol feedback (resistance to suppression after DST), as post-DST was correlated with 08:00 pm salivary cortisol in most study groups (all but CKD stages I–II). In our study, CBG, albumin and total protein levels were similar between study groups, ruling out a difference in cortisol binding, similar to previous findings (5).

Hair cortisol remained unchanged across CKD stages. This suggests that cumulative exposure to cortisol is not increased in this population, despite the dynamic changes in the HPA axis. This contrasts with a previous study in haemodialysis subjects that suggested an increased cortisol exposure over the day indirectly estimated through serial measurements (serum or salivary cortisol) (4). In case of a minimal degree of hypercortisolism, it may be speculated that a higher amount of excess cortisol is needed to produce a significant accumulation in hair (29). The finding of a progressive increase in salivary cortisol AUC with decreasing kidney function in our study supports this hypothesis. HC is a measurement of mid and long-term exposure to cortisol (1 cm hair = 1 month). Its validity, reliability, and correlation with the classic biochemical tests of the HPA axis have been thoroughly studied in healthy volunteers, CS, and adrenal insufficiency (10–13, 15–17). We observed a correlation of HC with age, which was lost when adjusting for confounders. Previous studies are discordant, some have shown increased levels with increasing age whereas others have not (10, 17). HC does not significantly correlate with sex and smoking but correlated with alcohol consumption, which has also been described (19). Nevertheless, heavy alcohol consumption was an exclusion criterion in the study and the multivariate analyses remained unchanged when alcohol consumption was included.

The mechanisms leading to changes in the HPA axis in CKD remain unknown. On the one hand, an activation of the HPA axis is suggested. Evidence suggests that ACTH may have renoprotective effects, mainly through systemic immunomodulation and anti-inflammatory actions through the melanocortin pathways and protective effects on kidney cells, particularly podocytes, *via* the melanocortin and neurogenic pathways. It has been hypothesised that ACTH would be upregulated in initial phases of CKD, an initial adaptive response which later becomes inadaptative (30). In accordance with this notion, complications of advanced CKD such as metabolic acidosis, low-grade inflammation, and stress related to life with a chronic condition could be drivers for a greater pituitary secretion of ACTH (25). On the other hand, a decreased intracellular inactivation of cortisol by the 11β-hydroxysteroid-dehydrogenase (HSD)-2 with decreasing renal function is hypothesised. This enzyme, which is highly expressed in the kidneys, inactivates cortisol to cortisone and prevents an indiscriminate activation of the mineral corticoid receptor by cortisol. A decrease in 11β-HSD-2 activity would lead to cortisol activating of the mineralocorticoid receptor causing adverse outcomes in the renal and cardiovascular systems and influence the rates of cortisol appearing in the systemic circulation (31). Reduced activity of the 11β-HSD-2 has been described in CKD (including patients on haemodialysis), T2D, advanced age, and inflammation settings (30, 32, 33). Another possible mechanism is an increased half-life of cortisol when kidney function is impaired leading to higher levels of cortisol in circulation, which translates into changes in cortisol rhythm and inadequate DST (28). Finally, little research in haemodialysis subjects suggests an impaired renal clearance of cortisol (accumulation of cortisol metabolites) (34) and a less effective hepatic cortisol metabolism (an accumulation of end products of cortisol hepatic metabolism) (35). Data regarding these mechanisms in earlier CKD stages are lacking.

In our cohort, cortisol after DST was linearly and inversely correlated with eGFR. Moreover, cortisol after DST and glucose were independently associated with an eGFR <30 mL/min/m^2^, independently of CKD duration or other cardiovascular risk factors. Olsen et al. found eGFR 30–60 (together with age and adenoma size) to be the main predictor of a cortisol post-DST >1.8 mcg/dL in subjects with adrenal incidentalomas (6). In another study in subjects with T2D, cortisol after DST was negatively correlated with eGFR and positively with CKD markers. Furthermore, higher terciles of cortisol after DST (as early as cortisol post-DST 0.6 mcg/dL) were associated with an increased prevalence of CKD, suggesting an activation of the HPA axis as a risk factor for the development of CKD in T2D (36). One study indirectly assessed the 11β-HSD system, describing a dysregulation toward increased intracellular cortisol in T2D, which was more pronounced in subjects with CKD (31). Finally, a recent study concluded that the 11β-HSD-mediated glucocorticoid activation in T2D is determined by inflammation (higher CRP) and is associated with diabetes and poorer glycaemic control in patients with CKD stages III–V (37). In a study in a cohort with hypertension, serum cortisol (8:00 h am) was negatively associated with eGFR and positively associated with CKD markers and higher cortisol terciles were associated with worse renal function (38).

In our cohort, a stepped increase in cardiometabolic comorbidities with CKD severity was observed (hypertension and dyslipidaemia prevalence, glucose, insulin, HOMA-IR, and triglycerides). A statistically significant linear trend was observed for CVD, although only three subjects had had a cardiovascular event (one in stage III and two in stage IV CKD). This finding suggests a higher cardiovascular risk in moderate and advanced CKD. One previous study found an association between serum cortisol before the dialysis session (8:00 h am–12:00 h pm) with mortality at 20 months in haemodialysis patients. In addition, cortisol correlated with CRP, suggesting a relation with inflammation (39). The sum of 24-h urinary cortisol metabolites was positively associated with systolic BP in a cohort of subjects with CKD stages II–III (40). There are no studies regarding the relation between cortisol and cardiovascular disease or mortality in pre-dialysis CKD.

When analysing the relationship of changes in the HPA axis with metabolic comorbidities, we describe an independent positive correlation between HC and VAT volume. In some studies, increased HC was correlated with features of the metabolic syndrome (especially BMI, abdominal obesity, and HbA_1c_) (18) whereas others found association only with CVD and T2D but not with BMI, WC, or smoking (19). Higher HC was reported in elderly people admitted for myocardial infarction compared with admissions for other causes; these also had higher BMI and LDL-c and lower HDL-c (34).

Regarding HC and body composition, the results of previous studies are conflicting. Some found a positive association between HC and BMI in men (18), whereas others found no relationship between HC and BMI in young (41) or elderly subjects (19). No previous studies have assessed HC and body composition in CKD. Our study includes subjects within a wide age range (52.9 ± 12.2 years), including elderly subjects (the eldest 77.4 years old). Both in older age (42) and CKD (37), changes in body composition occur, with an increase in body fat and a decrease in fat-free mass despite a stable BMI. We describe an independent correlation of HC with VAT volume, which better assesses fat mass in CKD (see above). To sum up, it cannot be ruled out that a mild hypercortisolism in CKD could contribute to an enlarged VAT volume, which in turn is associated with an adverse metabolic phenotype and increased cardiovascular burden (43).

Several limitations should be acknowledged. First, its observational design precludes drawing conclusions on causality. Second, the inclusion criteria were strict, so it may not be representative of the general CKD population and hamper generalizability. Third, the study does not include positive controls (CKD patients with hypercortisolism). Fourth, HC measurements were performed by immunoassay, which may present cross-reactivity with other structurally related molecules, such as cortisone. However, the immunoassay used presented adequate limit of quantification for the expected concentrations in hair and the cross-reactivity with other steroids is low, especially with cortisone (0.13%). Finally, while we employed a widely used (9, 14, 16) and well-validated (24, 41) procedure for hair cortisol measurements, enhanced cortisol extraction from hair samples can be achieved by hair grinding in conjunction with sequential and alternating solvents, rather than relying on hair mincing and a single methanol extraction (10). In fact, there is currently an urgent need to standardise procedures for cortisol extraction and quantification in hair, since various factors can impact their reliability including hair characteristics, the methods used for hair sample collection and storage, sample processing, and analytical techniques (10).

Based on our findings, there are several avenues for future research. First, prospective studies monitoring cortisol exposure over time could shed new light on the progression of HPA axis changes and their relation with CKD progression. Likewise, the physiopathological mechanisms underlying HPA axis disruption in CKD and its association with body composition and kidney damage need to be further explored, especially in initial phases. In addition, future trials could explore the effectiveness of interventions aiming to restore normal cortisol homeostasis (such as peripheral glucocorticoid activation blockade) and the impact on CKD progression and vice versa.

In summary, we describe changes in cortisol excretion, circadian rhythm, and dynamic study of the HPA axis: namely, a blunted diurnal decline and an impaired negative feedback regulation, in contrast to unchanged hair cortisol concentrations in subjects with CKD stages I to IV. Hair cortisol was independently associated with VAT volume, whereas cortisol after DST was associated with severe kidney damage. This is the first study to perform a thorough study of the HPA axis in CKD including hair cortisol concentrations and its association with metabolic comorbidities. Nevertheless, further studies are needed to investigate to a greater extent the sensitivity and reliability of hair cortisol as a tool for the diagnosis of Cushing syndrome in patients with impaired kidney function.

## Data availability statement

The original contributions presented in the study are included in the article/**Supplementary Material**. Further inquiries can be directed to the corresponding authors.

## Ethics statement

The studies involving humans were approved by CEIM Hospital Clinic Barcelona. The studies were conducted in accordance with the local legislation and institutional requirements. The participants provided their written informed consent to participate in this study.

## Author contributions

LB: Conceptualization, Data curation, Formal analysis, Investigation, Methodology, Software, Visualization, Writing – original draft, Writing – review & editing. AV-B: Formal analysis, Methodology, Software, Visualization, Writing – review & editing, Data curation, Investigation. MB: Data curation, Formal analysis, Investigation, Methodology, Writing – review & editing, Validation. LQ: Data curation, Formal analysis, Investigation, Writing – review & editing, Conceptualization, Supervision. GR: Data curation, Formal analysis, Investigation, Writing – review & editing, Methodology, Visualization. DD-C: Data curation, Formal analysis, Investigation, Methodology, Writing – review & editing. CV: Data curation, Formal analysis, Methodology, Writing – review & editing, Conceptualization, Validation. MC: Data curation, Formal analysis, Writing – review & editing, Investigation, Writing – original draft. MM: Formal analysis, Writing – review & editing, Conceptualization, Methodology, Project administration, Validation, Visualization. AA: Formal analysis, Methodology, Validation, Visualization, Writing – review & editing, Investigation, Software, Supervision. GC: Methodology, Software, Supervision, Validation, Visualization, Writing – review & editing, Conceptualization, Data curation, Project administration, Resources. FH: Conceptualization, Methodology, Project administration, Resources, Software, Supervision, Validation, Visualization, Writing – review & editing, Formal analysis, Funding acquisition.
