# Supplementary material for: Hair cortisol and changes in cortisol dynamics in chronic kidney disease
Source: Front Endocrinol (Lausanne). 2024 Mar 25;15:1282564. doi: 10.3389/fendo.2024.1282564 (PMC11024788; doi:10.3389/fendo.2024.1282564)
Supplement: Supplementary file 1 [file DataSheet_1.pdf]

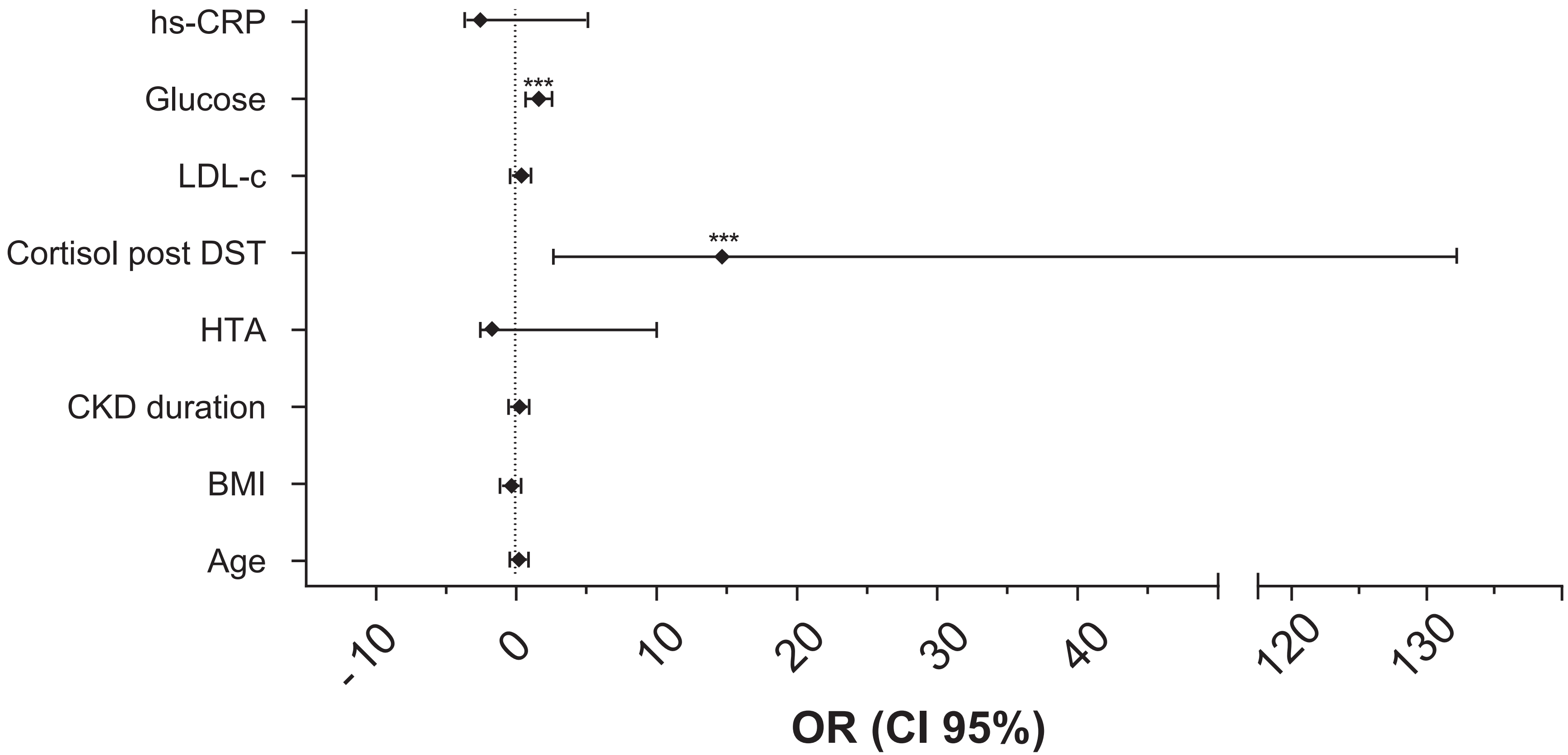

Supplementary Figure 1. Cortisol after DST independently associated with an eGFR <30 mL/min/m^2

**BMI: body mass index; hs-PCR: high sensitive C-reactive-protein, HTA: hypertension, LDL-c: low-density lipoprotein cholesterol; DST: dexamethasone suppression test, CKD: chronic kidney diseases, eGFR: estimated glomerular filtration rate.*

Parameter	Pearson correlation	Degree of association with VAT volume				
		Standardized coefficient	β	95% CI		p value
				LL	UL	
Hair cortisol	0,316	0,22	54,488	10,93	98,03	0,015
Sex	-0,412	-0,28	-56,1	-91,3	-20,3	0,003
BMI	0,593	0,505	123,1	79,464	166,781	<0.001

uded variables from the stepwise model

Parameter	Beta in	p value	Colinearity tolerance
Smoking	0,015	0,925	0,965
Age	0,075	0,665	0,773
eGFR	0,023	0,503	0,621

Model Summary				Change Statistics				
Model	R	R Square	Adjusted R Square	R Square Change	F Change	df1	df2	Sig. F Change
1	0,696	0,484	0,461	0,484	21,283	3	68	<0.001

a. Predictors: (Constant), Sex, Cort_hair, BMI

Supplementary Table 1.

**VAT: Visceral adipose tissue; BMI: body mass index; eGFR: estimated glomerular filtration rate.*

Factor	Pearson / Spearman correlation	p-value
Dyslipidaemia	0, 201	0,103
Cardiovascular disease	0,238	0,107
Hypertension	0,136	0,202
Depression	0,039	0,795
Waist-to-hip ratio	0,184	0,216
Glucose	0,170	0,254
HbA_{1c}	0,237	0,108
LDL-c	0,014	0,927
HDL-c	-0,151	0,312
AST	-0,187	0,208
ALT	-0,212	0,153
GGT	0,205	0,168
BR	-0,279	0,058
ALP	0,085	0,572
Proteins	-0,021	0,886
Albumin	0,202	0,173
Sodium	-0,083	0,580
Potassium	0,215	0,147
Osmolarity	0,243	0,079
Leucocytes	0,192	0,197
Haemoglobin	-0,131	0,380
Platelets	0,162	0,278
TSH	0,125	0,403
FT4	-0,233	0,115

ALP:Alkaline phosphatase; ALT: alanine transaminase; AST: aspartate aminotransferase; BR: total bilirubin; FT4: thyroxine; GGT: Gamma-glutamyl transferase; HDL-c: high-density lipoprotein cholesterol; LDL-c: low-density lipoprotein cholesterol; PTH: parathormone; TSH: thyroid stimulating hormone.

Supplementary Table 2. Correlation of variables and serum cortisol levels after 1mg dexamethasone test in the whole cohort
